# Targeting SARS-CoV-2 Variants with Nucleic Acid Therapeutic Nanoparticle Conjugates

**DOI:** 10.3390/ph14101012

**Published:** 2021-10-01

**Authors:** Hanah F. Huber, Majid Jaberi-Douraki, Sarah DeVader, Cesar Aparicio-Lopez, Juliet Nava-Chavez, Xuan Xu, Nuwan Indika Millagaha Gedara, Natasha N. Gaudreault, Robert K. Delong

**Affiliations:** 1 Nanotechnology Innovation Center, Department of Anatomy and Physiology, College of Veterinary Medicine, Kansas State University, Manhattan, KS 66506, USA; hhuber@vet.k-state.edu (H.F.H.); sdevader05@ksu.edu (S.D.); cesar03@ksu.edu (C.A.-L.); julietn@ksu.edu (J.N.-C.); 2 1DATA Consortium and Department of Mathematics, Kansas State University Olathe, Olathe, KS 66061, USA; jaberi@ksu.edu (M.J.-D.); xuanxu@ksu.edu (X.X.); mgnindika@ksu.edu (N.I.M.G.); 3 Department of Diagnostic Medicine/Pathobiology, College of Veterinary Medicine, Kansas State University, Manhattan, KS 66506, USA; nng5757@vet.k-state.edu

**Keywords:** SARS-CoV-2, nanoparticle, TFO, variant, homopurine, palindrome

## Abstract

The emergence of SARS-CoV-2 variants is cause for concern, because these may become resistant to current vaccines and antiviral drugs in development. Current drugs target viral proteins, resulting in a critical need for RNA-targeted nanomedicines. To address this, a comparative analysis of SARS-CoV-2 variants was performed. Several highly conserved sites were identified, of which the most noteworthy is a partial homopurine palindrome site with >99% conservation within the coding region. This sequence was compared among recently emerged, highly infectious SARS-CoV-2 variants. Conservation of the site was maintained among these emerging variants, further contributing to its potential as a regulatory target site for SARS-CoV-2. RNAfold was used to predict the structures of the highly conserved sites, with some resulting structures being common among coronaviridae. An RNA-level regulatory map of the conserved regions of SARS-CoV-2 was produced based on the predicted structures, with each representing potential target sites for antisense oligonucleotides, triplex-forming oligomers, and aptamers. Additionally, homopurine/homopyrimidine sequences within the viral genome were identified. These sequences also demonstrate appropriate target sites for antisense oligonucleotides and triplex-forming oligonucleotides. An experimental strategy to investigate these is summarized along with potential nanoparticle types for delivery, and the advantages and disadvantages of each are discussed.

## 1. Introduction

The COVID-19 pandemic has caused a global public health crisis. Although current vaccines can be greater than 90% effective at preventing severe disease, there is still grave concern over the emergence of variants which may circumvent currently available vaccines and antiviral drugs still in the research and development phases. Recently, antisense, aptamer, and RNA-based drugs have been clinically approved, with promising preclinical data on the safety of antisense oligonucleotide (ASO) conjugates [1,2]. Successful attempts in targeting SARS-CoV-2 with nucleic acid therapies delivered by a nanoparticle have been documented, the most notable of which are the Pfizer and Moderna mRNA vaccines. Most studies attempting to target the virus with nanomaterials or nanotechnology have employed lipid or small protein nanoparticles as the delivery vehicles or were attempting to increase the cellular uptake of nanoparticles [3,4,5,6,7]. Comparatively, very little work has been conducted on exploring the effect of synthetic inorganic nanoparticles in targeting SARS-CoV-2 through the delivery of nucleic acids. Studies which did explore the effects of inorganic nanoparticles typically used nanoparticles composed of gold and mesoporous silica nanoparticles, with very few groups looking at more biologically compatible nanoparticles [5,6,8]. In an attempt to address this, physiologically based nanoparticle metal compositions have recently been developed. These physiometacomposite (PMC) nanoparticles can form conjugates to ASOs which are biocompatible and active for delivery into cells [9]. In order for these conjugates to be effectively utilized, a target site in the viral genome must first be identified. In this work, an RNA genome-wide analysis was conducted to identify potential regulatory sites within the SARS-CoV-2 genome which could serve as target sites for ASO or other types of nucleic acid drugs. The structure of these potential regulatory or target sites was predicted and compared in SARS-CoV-2 variants of concern, followed by a discussion over how to target and deliver ASO or TFO (triplex-forming oligonucleotide) conjugates to these sites with various nanoparticle candidates.

## 2. Results

**Figure 1:**
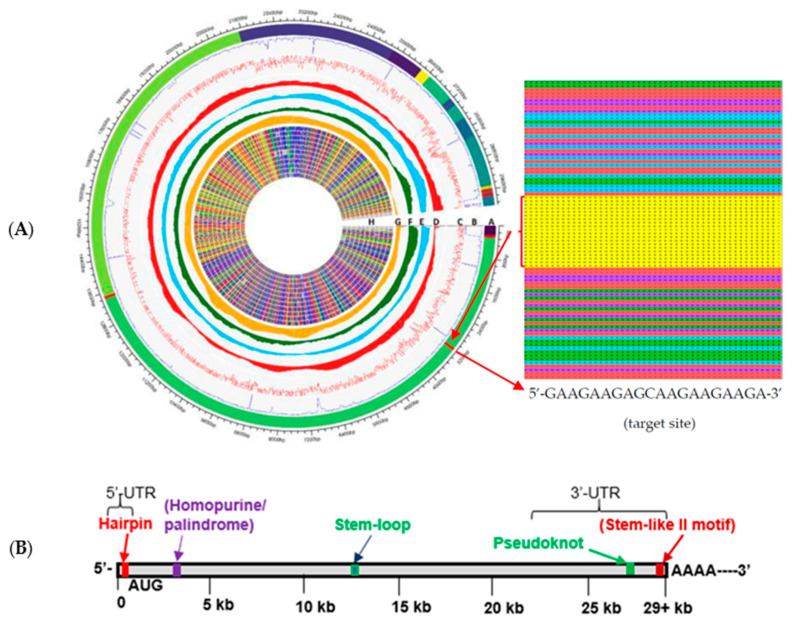
(**A**)**:** GWRPD map of SARS-CoV-2. Track A: multiple (ORFs) indicating different NSARS-CoV-2 ORF1ab polyproteins, 5′ UTR and 3′ UTR. Red stripes in these regions represent the five most conserved oligomer sequences. Track B: reinforcement learning approach with a comparison between the sparse-spike fragments of variant locations and highly conserved. Track C: red noise curve representing a similar approach for 311 SARS-related coronavirus isolates from 2003 to early 2019, includings mostly human hosts and several other species. Tracks D–G: Four tracks of red, blue, green, and orange histograms show the average distributions of A, C, G, and T nucleotides in all 1557 SARS-CoV-2 records. Track H: Heatmap of 12 sample sequences from 1557 sequences, mapped to the reference genome showing their conserved aligned regions and variations in some regions. Zoomed inset panel shows part of the complete alignment of 1557 with triplex target sequence and structure. (**B**): Mechanisms of RNA-level control predicted for SARS-CoV-2 based on RNA sequence conservation.

A genome-wide RNA profiling and differential analysis (GWRPD) of sequence variations in 1557 different variants of SARS-CoV-2 was performed. This analysis resulted in the identification of five sites with 98–99% conservation among all analyzed variants, as indicated by the red segments in the outer ring of the GWRPD map (Figure 1A).

Parallel bioinformatic predictions identified a priori potential G-C/A-T contents in the percentage bases for SARS-CoV-2 (Figure 1A (D–G)). The independent evaluation of sparse-spike fragments and highly conserved regions using both progressive and Thompson-Higgins-Gibson (THG) approaches reflects the high probability that these regions are well-maintained to characterize the specific RNA regulatory structures, help study their biomolecular interactions, delivery, and impact on RNA stability and activity (Figure 1A, zoomed inset panel).

Based on the above GWRPD analysis, a potential regulatory map of RNA-level molecular control of the SARS-CoV-2 virus was generated (Figure 1B). Four highly conserved regions, the predicted structures they form, and the location of each within the genomes of the 1557 variants included in the comparative analysis are indicated by the red and green segments and fonts in Figure 1B. Of primary interest is the homopurine/palindrome sequence indicated by the purple segment and font, located near the beginning of the genome within the coding region. Such homopurine/homopyrimidine palindromic sites often serve regulatory roles and may also hold roles in RNA-level control of the function and activity of the virus.

Interestingly, although some of the GWRPD-identified conserved sites could be anticipated based on earlier coronavirus studies, the most highly conserved site (99.9%) was the homopurine/palindrome site, which was previously unidentified [10]. With a sequence of 5′-GAAGAAGAGCAAGAAGAAGA-3′, this site is not a true palindrome due to the presence of an intervening cytosine in the middle of the sequence. This interruption makes the sequence a partial homopurine palindrome. Although other partial homopurine and homopyrimidine sites can be found throughout the genome of SARS-CoV-2, none demonstrate the same level of conservation as the one identified by the GWPRD analysis (Table S1, Supplementary Materials). An intervening cytosine in an otherwise perfect homopurine sequence has been successfully targeted with a TFO containing the base analog 8-oxoadenine (8-oxo-A) in the context of a methylphosphonate oligodeoxyribonucleoside, albeit to dsDNA [11]. Use of methylphosphonate chemistry in the backbone of the TFO means it is less prone to non-specific protein binding and nuclease digestion [12,13]. Additionally, Kierzek et al. were recently successful in targeting a similar homopurine/homopyrimidine stem–loop in Influenza A virus with TFO [14,15]. The target site, as well as the other four 98–99% conserved sites identified by the GWRPD analysis which can be targeted by TFO or ASO, are summarized in Table 1.

**Figure 2:**
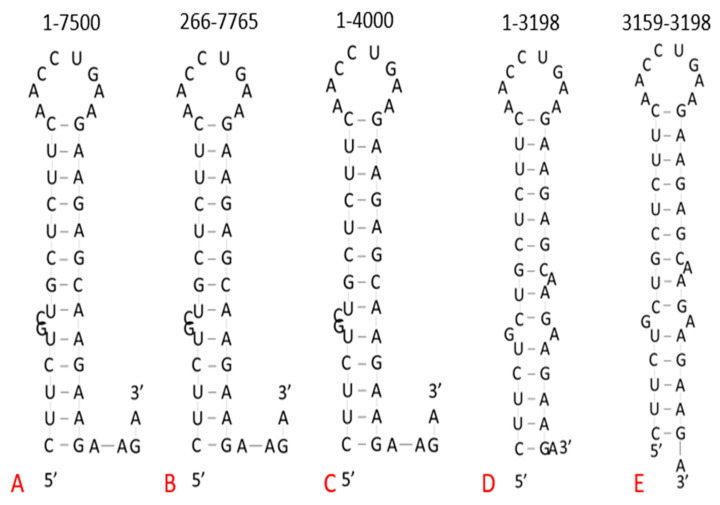
Comparison of homopurine/palindrome structure using different regions of the genome in RNAfold WebServer. (**A**) Predicted structure when folding nucleotides 1–7500, the maximum length allowed by RNAfold. (**B**) Predicted structure when folding from the end of the 5′ UTR and including the maximum number of nucleotides allowed by the program. (**C**) Predicted structure when folding nucleotides 1–4000, a short stretch beyond the end of the palindromic region. (**D**) Predicted structure when folding from the beginning of the genome to the last nucleotide of the palindromic sequence. (**E**) Predicted structure of the isolated palindromic sequence.

The predicted structures produced by RNAfold are shown in Figure 2. Folding the first 7500 nucleotides of the SARS-CoV-2 genome produced a stem–loop structure, as indicated in the results of the comparative analysis (Figure 1B). This structure was used as a reference to compare predicted structures using different regions or shorter segments of the genome in the program. Additionally, the isolated stem–loop sequence was folded by RNAfold to verify the integrity of the structure, as this isolated sequence could be used in further in vitro studies regarding this site. Regardless of the amount of the genome folded and which regions were included, RNAfold consistently predicted a stem–loop structure for the highly conserved target site (Figure 2).

### 2.1. Role of RNA Structures in the Function of Coronavirus

Coronaviruses are highly structured and a significant number of secondary and tertiary RNA structures have been identified, not only in the untranslated regions (UTRs), but throughout the coding regions of the genome [16,17,18]. At least eight major stem–loop structures have been identified in the 5′-UTR of coronaviruses, of which at least three are highly conserved among coronaviruses and play roles in replication, sgRNA synthesis and translation [19,20,21,22,23]. The ribosomal frame-shifting element (FSE), consisting of an RNA pseudoknot structure at the junction of ORF1a/b, is essential for coronavirus replication and is one of the best characterized of these structures [24,25]. Structures have also been identified in the 3′-UTR, of which some have been found functionally relevant in at least some betacoronaviruses [23,26,27,28,29].

Although most remain uncharacterized, the highly conserved nature of these RNA structures among coronaviruses suggests that they may have functional relevance, perhaps through ensuring genomic stability or long-range RNA–RNA interactions or RNA–protein interactions important for viral replication [16,17,18,26]. Genomic structure modeling studies indicate that the SARS-CoV-2 genome forms more short-range stable secondary structures than long-distance interactions compared to other RNA viruses, which may be important for viral genome stability and replication fidelity [17,18,26].

The region of interest identified here, referred to as the target site, is located in the 5′-end coding region of ORF1a/b and the nsp3 gene. ORF1a and ORF1b share the same 5′ start codon, but the FSE partially disrupts recognition of the stop codon, resulting in the replicase polyproteins, pp1a and pp1ab [25]. ORF1a and ORF1b encode immediate early and early proteins that include innate immune antagonists and those involved in viral transcription and replication. Together, ORF1a and ORF1b produce non-structural proteins, such as nsp1-16. Nsp3 is a papain-like protease responsible for N-terminal cleavage of the replicase polyprotein, is involved in the assembly of viral replication complexes, and inhibits type I interferon induction, among other functions [30,31,32]. Thus, targeting this highly conserved structure sequence described here could have important implications related to viral replication and/or inhibition of the host’s innate immune response.

### 2.2. Target Site in Variants of Concern

The specific variants, target site sequence, and the loci at which they begin are shown in Table 2. The target site sequence was 100% conserved among the variants compared, which was to be expected based on the results of the more in-depth GWRPD analysis from the beginning of this paper. It is worth noting that this sequence was located at the exact same locus compared to the Wuhan reference sequence in half of the variants examined. Those that were not an exact match were located a maximum of 135 nucleotides earlier in the genome, although this was not a large enough difference to place the target site outside of the coding region of the virus.

**Figure 3:**
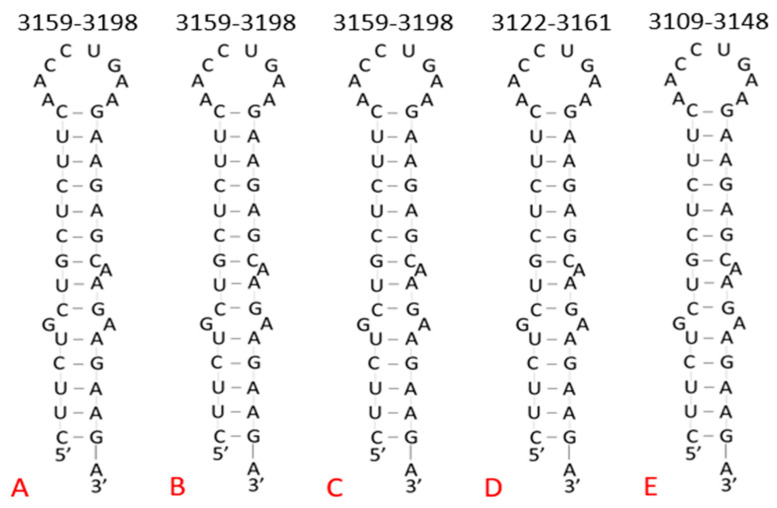
Comparison of isolated palindromic target sequence in variants of concern using RNAfold WebServer. The structure from the Wuhan sequence (**A**) served as the reference for the variants from Brazil (**B**), the United Kingdom (**C**), South Africa (**D**), and India (**E**). The structures are identical with no differences in the sequence, overall stem–loop structure, and location of bulges within the straight regions of the structure.

Furthermore, the isolated target sequence within the previously mentioned variants of concern from Brazil, the United Kingdom, South Africa, and India were folded using RNAfold. The second variants from each region in Table 2 were folded, and the predicted structures were compared (Figure 3). Each of these predicted structures were identical to the Wuhan reference sequence, again supporting the regulatory significance of maintaining this site. The only notable difference was that the South African and Indian variants had stem–loop structures beginning 37 and 50 nucleotides earlier than the reference sequence, respectively. However, this small difference of location is not significant because the structure is still located within the coding region of the viral genome and forms a stem–loop structure identical to the Wuhan reference sequence, indicating that it can still be targeted with the proposed TFO delivery system.

## 3. Discussion

**Figure 4:**
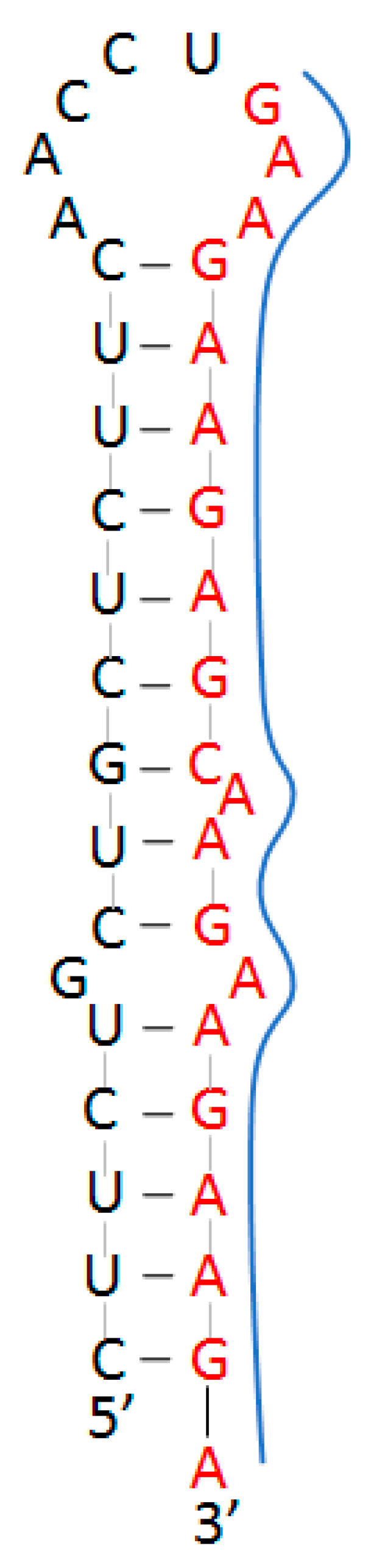
SARS-CoV-2 TFO (blue) target site. The predicted stem loop of the synthetic isolated sequence containing the homopurine site (red nucleotides) will be targeted by a synthetic TFO (blue line), which will bind to the target site through complementary base pairing.

In an effort to determine the relative importance of the stem-loop structure, specifically the partial homopurine palindrome site, in regulating the activity of SARS-CoV-2, this site was targeted by TFO. As mentioned previously, Kierzek et al. targeted a similar stem-loop in Influenza A virus with TFO, which resulted in an antiviral effect demonstrated by cell culture assays [14,15]. Regarding SARS-CoV-2, the predicted structure of the target site provided by RNAfold had about 13 nucleotides involved in the stem of the structure. The 3′ end had one to four free bases, depending on the length of the sequence being folded, and the 5′ end did not contain free bases. With longer regions run through the folding program, the non-palindrome sequence contained a GC-bulge and the palindrome sequence was linear (no bulges). However, when folding shorter regions such as the isolated target sequence, the predicted structure changed. The non-palindrome sequence became a G-bulge and the palindrome sequence developed two A-bulges. These bulges may serve to increase the space in which the TFO can enter this region of the viral genome and bind to the palindrome sequence via U-A-U and C-G-C base triplets (Figure 4). Stabilization of the triplex formation can be achieved through the use of 5-methylcytosine (5Me-C) and deoxyuridine (dU) in TFO to target the G-C and A-U base-pairs, respectively, as previously reported [33].

### 3.1. Target Site Characterization

The target site can be characterized using techniques commonly used to determine RNA structure, including nuclease mapping, circular dichroism (CD) spectroscopy, and UV thermal melting of the isolated sequence. Nucleases capable of cleaving bases not involved in a double-stranded segment of the RNA sequence, such as those involved in the bulges and loop, can be used to digest the stem–loop structure. The produced segments can then be separated and identified through the use of gel electrophoresis. This will produce a unique band pattern in the gel which can be used to identify the presence of specific structures within the target site. The structures identified by the digest can then be compared with the original RNAfold-predicted structure. The band pattern obtained from this experiment will then serve as a control for future experiments involving the target site. The other proposed characterization methods to determine the structure of the isolated target site in vitro involve obtaining the UV melting curve and CD spectrum through the use of a SpectraMax i3x Multi-Mode Plate Reader and CD spectrometer, respectively. The shape of the produced melting curve can provide information on the stability of the structure, G-C content, reversibility of the melting process, and presence of the secondary structure within the sample [34]. Additionally, the melting point of the isolated sequence will serve as a control for further experiments exploring the interaction of the target site with other molecules. The CD spectrum of the isolated target sequence will provide information on the secondary structure under different temperature and salt conditions. This will be useful in determining what structure the target sequence will most likely form under physiological conditions. The spectrum of the isolated sequence in the absence of other molecules can then be used as a reference to identify changes in structure after exposure to or interaction with other molecules

### 3.2. Confirmation of Triplex Formation

Upon characterization of the target site, the proposed triplex-forming region of the target site was targeted using a nanoparticle-delivered TFO. To determine whether the TFO was able to bind to the target site as predicted and to provide insight to optimal conditions which promote triplex formation, CD spectra, thermal melting points, and gel electrophoresis gel shift assay were performed and compared to data collected from target site characterization and any pre-existing literature. Each of these techniques are commonly used in RNA interaction studies.

CD spectra and thermal melting points were obtained through the use of a CD spectrometer and SpectraMax i3x Multi-Mode Plate Reader, respectively. CD spectra of the target site and TFO as well as of the target site and TFO-nanoparticle conjugate were collected under the same conditions used to characterize the structure formed by the target site alone. Significant changes in either the location or intensity of the peaks of the CD spectra of the target site in the presence of TFO from that of the target site in the absence of TFO confirmed interaction between the two, a concept previously demonstrated by Kierzek et al. [15]. From there, CD spectra were collected under different temperature conditions. Any triplex base pairs (complementary bonds between nucleotides within the triplex and target site) should dissociate in a temperature-dependent manner. This dissociation is detected by the CD spectrometer, and changes in spectra can indicate at what temperatures the TFO (if bound) dissociates from the target site and the target site dissociates into ssRNA. Just as changes in CD spectra can indicate changes in sample structure, changes in the thermal melting point of the target structure after exposure to the TFO can be used to indicate triplex formation. Thermal melting point data also provide information on the stability of the triplex once formed, which was anticipated to be more stable than the predicted stem–loop and thus have a higher melting point.

Gel electrophoresis can be used to determine optimal ratios which promote TFO binding, and thus, triplex formation. The target structure in the absence of TFO was used as a control to establish the standard band location within the gel. The band pattern of samples containing both the target structure and TFO at varying concentrations can be used to determine whether the TFO was able to interact with and bind to the target structure (indicated by a band shift closer to the wells) or not (no band shift from control). Additionally, the information provided by the gel can be used to determine the binding affinity of the TFO to the target site [15]. Nuclease digestion followed by gel electrophoresis can also be used to confirm triplex formation. Nucleases which cleave at single-stranded sites within the RNA (such as the bulges and loop of the target site structure) have fewer sites at which they can be active within the TFO-bound target site. The resulting digested triplex was anticipated to have fewer nucleotides removed than the stem–loop of the target site, producing a band with a smaller R_f_ in the gel compared to the band from nuclease digestion of the target site in the absence of TFO.

### 3.3. Nanoparticle-Mediated Delivery

As previously mentioned, nucleic acids can be targeted using ASO and TFO methods. However, due to their instability, free nucleic acid therapeutics are not available in the absence of a nanoparticle stabilizer or carrier. Another method to target nucleic acids involves the use of aptamers. Aptamers can bind proteins and nucleic acid sequences with high selectivity and strong interactions. This technology can be applied in diagnostics, drug delivery systems, and therapeutics. Depending on the protein targeted by the aptamer, these molecules can have a wide range of applications. Aptamers can increase the selectivity of an antiviral particle or possess antiviral activity itself. The aptamer IBRV-A4 was able to prevent infection of bovine herpesvirus 1 in cell culture trials by preventing cellular uptake of the virus [35]. However, this technology can be hindered by biological and physical barriers that can degrade or damage the genetic material, such as high renal clearance, susceptibility to serum nucleases, uptake by phagocytes, and induced immune responses [36,37]. Nanoparticles (NPs) can help surmount many, if not all, of these barriers.

Lipid-based and polymer-based nanoparticles are often used as vehicles to deliver aptamers because they can transport large payloads of charged molecules (e.g., aptamers) through the cell membrane using cell-penetrating peptides [38,39,40]. NPs can ensure a long and constant release of an aptamer to maintain a desired concentration at the target site and guarantee aptamer-protein interactions. Previously, an siRNA target formulation of an ultra-conserved sequence of the hepatitis C virus was tested to determine binding and regulation capabilities of this molecule. Through the use of nanosomes, the siRNA of the study had a 100% delivery efficiency in cell culture and demonstrated the ability to regulate the ribosome entry site for translation [39]. There are FDA-approved materials, such as PLGA, which possess better physiological stability and encapsulation efficiency. Although these materials have not yet been tested against a virus, they have been proven to increase the stability of aptamers. Other NP candidates for aptamer stabilization and delivery are inorganic NPs. Inorganic NPs are commonly used as imaging probes due to their fluorescence capabilities, but they also are used to stabilize aptamers and for the delivery of genetic material. Gold-aptamer probes have been shown to possess high selectivity and sensitivity [41]. Early on, gold nanoparticle siRNA conjugates showed delivery into the brain with preclinical efficacy against glioblastoma [42]. More recently, manganese zinc sulfide (MnZnS) nanoparticles have demonstrated antiviral activity in the unconjugated form, and anticancer activity against drug-resistant cancer when complexed with an ASO [8]. Targeting of inorganic nanoparticles can also be improved by adding an aptamer to the nanoparticle’s surface.

TFOs can be conjugated to the surface or incorporated into the core of NPs and, in combination with other ligands, can improve the stability and targeting capabilities of the NP conjugate. Each of the previously discussed nanoparticle delivery methods is summarized in the context of TFO and stem-loop delivery in the graphical abstract at the beginning of this paper. As shown in the graphical abstract, delivering either the TFO or stem–loop structure to a cell infected with SARS-CoV-2 can potentially inhibit further viral activity, especially in the context of an antiviral nanoparticle such as MnZnS. Synthetic stem-loop structures can act as decoys for viral and host proteins, limiting those available to interact with the fully intact viral genome. Alternatively, TFOs can be delivered to virally infected cells, with the potential to form a triplex with the target site stem–loop in the SARS-CoV-2 genome and interfere with the genome’s natural folding in vivo.

## 4. Methods

### 4.1. GWRPD Analysis

To achieve the data visualization shown in Figure 1 for on-going genetic surveillance and mutational analysis, the NCBI Virus Sequences for Discovery database was used to categorize available data related to coronaviruses, including SARS-CoV, which has a complete genome and high sequence similarity to SARS-CoV-2 and is the best-characterized coronavirus. The data used in this study have been curated into our databank in 1DATA (www.1DATA.life)—was last accessed 15 July 2021 –and then aligned in MATLAB (R2020, Math-Works), MAFFT (Berkeley Software Distribution), and MEGA (Pennsylvania State University) for multiple sequence alignment and Bayesian inference phylogenetics from a reinforcement learning approach using a progressive method and the Thompson–Higgins–Gibson (THG) method [43,44,45]. The RNA sequence data included 1557 records of isolates from the oronasopharynx (730), swab (196), lung (22), feces (4), blood (1), urine (1), and saliva (603). Of those included in the analysis, the majority of records were from human hosts (over 1550), with some animal hosts represented, including *Canis lupus familiaris* (1), *Felis catus* (1), *Mustela lutreola* (7), and *Panthera tigris jacksoni* (1) [46]. The structure of these SARS-CoV-2-specific RNA sequences, their interaction and targeting to and by ASO, TFO, nanoparticle delivery, and sequence conservation has never been studied before.

### 4.2. RNA Folding

Due to its high conservation among variants, the structure of the partial homopurine palindrome site was explored using the RNAfold WebServer online program. The program is capable of folding up to 7500 nucleotides at a time. The target site was located between nucleotides 3179 and 3198; therefore, the genome was not folded past nucleotide 7765. Multiple folds were performed, using different lengths and regions of the SARS-CoV-2 genome, with the focus being placed on the region surrounding the target site.

### 4.3. Target Site for Variants of Concern

To further demonstrate its potential significance, the sequence of the partial homopurine palindrome target site was compared in SARS-CoV-2 variants of concern identified by the Centers for Disease Control and Prevention: the alpha variant (B.1.1.7), which originated in the United Kingdom; the beta variant (B.1.351), which originated in South Africa; the delta variant (B.1.617.2), which originated in India; and the gamma variant (P.1), which originated in Brazil. Each of these variants has been a source of increasing concern on a national and global scale as they have spread outside their country of origin, further exacerbating the effects of the pandemic. The Wuhan isolate served as the reference sequence for this comparison. Isolate sequences for each variant and the Wuhan reference isolate were obtained through the NCBI SARS-CoV-2 Nucleotide Records provided by the National Institutes of Health. The searches were performed periodically during the months of May 2020 through July 2020. Of the isolates included in this paper, collection dates ranged from December 2019 (GenBank accession number NC_045512.2) to June 2021 (GenBank accession number MZ558086.1).

## 5. Conclusions

Although recent vaccines have proven effective in helping to counteract the SARS-CoV-2 pandemic, variants have emerged, resulting in concern regarding the ability of the virus to develop resistance to available vaccines and antiviral drugs in development. RNA-targeted nanomedicines are a potential solution to this problem. Recently, a partial homopurine palindrome site within the coding region of the SARS-CoV-2 genome has been identified. GWRPD analysis showed that this site has a greater than 99% conservation among more than 1500 variants, including those which have recently emerged and demonstrated high infectiousness in the regions of Brazil, the United Kingdom, South Africa, and India. This site was further explored using RNAfold to predict the potential structure formed by this region of the genome in vivo. The resulting stem–loop structure can be targeted with a TFO delivered by nanoparticles. Depending on the nanoparticle used, the TFO can be stabilized and protected from the body while being delivered in high concentrations to target cells. However, before this approach can be explored, the target site structure must be characterized in order to more accurately understand how it interacts with the nanoparticle-TFO delivery system. Additionally, although targeting this unique structure with TFO is an attractive method, nanoparticle-mediated delivery of ASO or aptamer targeting this or other highly conserved sites are excellent alternative strategies to consider.

## 6. Patents

Disc. 2021-047; Our Docket No. 19484. mRNA SARS-CoV-2 vaccine construct. Inventors, Robert K. DeLong, Waithaka Mwangi and Juergen Richt. mRNA Vaccine Formulations and Methods of Using the Same. Kansas State University.

## Figures and Tables

**Table 1:** Highly conserved sites of the SARS-CoV-2 genome, as identified by GWRPD analysis.

Conserved Site (Base #)	% Conserved (Out of 1571 Variants)	Target Sequence (5′–3′)	Predicted Structure
Coding Region (3179–3198)	99.90%	GAAGAAGAG**C** ^1^ AAGAAGAAGAAGA	Homopurine, Palindrome Stem–loop
3′-UTR (29,721–29,761)	99%	UUCACCGAGGCCACGCGGAGUACGAUCGAGUGUACAGUGA	Hairpin
Coding Region (13,468–13,496)	99%	CGGUGUAAGUGCAGCCCGUCUUACACCG	Stem–loop
Coding Region (29,619–29,644)	98%	GGCCCACACTGGCTTTCCATTC	Pseudoknot
5′-Leader (231–265)	>97%	UCAUCAGCACAUCUAGGUUUCGUCCGGGUGUGACCGAAAGGUAA	Hairpin

^1^ The underlined base is the intervening cytosine which disrupts the otherwise homopurine sequence of the target site.

**Table 2:** Conservation of the target site among SARS-CoV-2 variants of concern.

Country and Variant (Base #)	Target Sequence ^1^ (5′–3′)	Sequence Length	GenBank Accession Number
Wuhan Reference (3179–3198)	GAAGAAGAG**C**AAGAAGAAGAAGA	20 nt	NC_045512.2
Brazil P.1 (3171–3190)	GAAGAAGAG**C**AAGAAGAAGAAGA	20 nt	MZ264787.1
Brazil P.1 (3179–3198)	GAAGAAGAG**C**AAGAAGAAGAAGA	20 nt	MZ169910.1
Brazil P.1 (3179–3198)	GAAGAAGAG**C**AAGAAGAAGAAGA	20 nt	MZ169911.1
UK B.1.1.7 (3179–3198)	GAAGAAGAG**C**AAGAAGAAGAAGA	20 nt	OU029086.1
UK B.1.1.7 (3179–3198)	GAAGAAGAG**C**AAGAAGAAGAAGA	20 nt	OU029131.1
UK B.1.1.7 (3179–3198)	GAAGAAGAG**C**AAGAAGAAGAAGA	20 nt	OU029144.1
Ghana B.1.351 (3179–3198)	GAAGAAGAG**C**AAGAAGAAGAAGA	20 nt	MW598408.1
South Africa B.1.351 (3142–3161)	GAAGAAGAG**C**AAGAAGAAGAAGA	20 nt	MZ376663.1
Djibouti B.1.351 (3125–3144)	GAAGAAGAG**C**AAGAAGAAGAAGA	20 nt	MZ520096.1
India B.1.617.2 (3153–3172)	GAAGAAGAG**C**AAGAAGAAGAAGA	20 nt	MZ558086.1
India B.1.617.2 (3129–3148)	GAAGAAGAG**C**AAGAAGAAGAAGA	20 nt	MZ340535.1
India B.1.617.2 (3154–3173)	GAAGAAGAG**C**AAGAAGAAGAAGA	20 nt	MZ558154.1

^1^ The intervening cytosine within the partial homopurine sequence was again underlined for each isolate.

## Data Availability

Data is contained within the article and supplementary material.

## Supplementary Materials

The following are available online at https://www.mdpi.com/article/10.3390/ph14101012/s1, Table S1: Partial homopurine and homopyrimidine sites within the SARS-CoV-2 genome.
