# Supplementary material for: Targeting SARS-CoV-2 Variants with Nucleic Acid Therapeutic Nanoparticle Conjugates
Source: Pharmaceuticals (Basel). 2021 Oct 1;14(10):1012. doi: 10.3390/ph14101012 (PMC8539335; doi:10.3390/ph14101012)
Supplement: Supplementary file 1 [file pharmaceuticals-14-01012-s001.zip › pharmaceuticals-1366647-supplementary.pdf]

Table S1. Partial homopurine and homopyrimidine sites within the SARS-CoV-2 genome.

Sequence Type (base #)	Target Sequence (5′-3′)	Sequence Length	Number of Mutations
**Homopurine (1801-1821)**	AAAAGGAAAAG**CT**AAAAAAGG	19 nt	2
**Homopurine (13610-13625)**	AAGAAAAGGA**C**GAAGA	15 nt	1
**Homopurine (29378-29399)**	AAAAAGGA**C**AAAAAGAAGAAGG	21 nt	1
**Homopyrimidine (21725-21742)**	TTCTT**A**CCTTTCTTTTCC	17 nt	1
**Homopyrimidine (25700-25717)**	CCCCTTTTCTCT**A**TCTTT	17 nt	1
**Homopyrimidine (26296-26313)**	CTTCTTTTTCTT**G**CTTTC	17 nt	1
